# Dynamic Reconstruction and Mesh Compression of 4D Volumetric Model Using Correspondence-Based Deformation for Streaming Service

**DOI:** 10.3390/s22228815

**Published:** 2022-11-15

**Authors:** Byung-Seo Park, Sol Lee, Jung-Tak Park, Jin-Kyum Kim, Woosuk Kim, Young-Ho Seo

**Affiliations:** Department of Electronic Materials Engeering, Kwangwoon University, Kwangwoon-ro 20, Nowon-gu, Seoul 01897, Republic of Korea

**Keywords:** 4D volumetric, remeshing, correspondence, dynamic reconstruction, mesh compression

## Abstract

A sequence of 3D models generated using volumetric capture has the advantage of retaining the characteristics of dynamic objects and scenes. However, in volumetric data, since 3D mesh and texture are synthesized for every frame, the mesh of every frame has a different shape, and the brightness and color quality of the texture is various. This paper proposes an algorithm to consistently create a mesh of 4D volumetric data using dynamic reconstruction. The proposed algorithm comprises remeshing, correspondence searching, and target frame reconstruction by key frame deformation. We make non-rigid deformation possible by applying the surface deformation method of the key frame. Finally, we propose a method of compressing the target frame using the target frame reconstructed using the key frame with error rates of up to 98.88% and at least 20.39% compared to previous studies. The experimental results show the proposed method’s effectiveness by measuring the geometric error between the deformed key frame and the target frame. Further, by calculating the residual between two frames, the ratio of data transmitted is measured to show a compression performance of 18.48%.

## 1. Introduction

There are various methods for producing a high-quality 3D model, but classical techniques have a problem because they require a lot of human resources and time to build a 3D model. A method of producing a 3D model can be classified into a manual sculpting method and an automated reconstruction method. Manually creating 3D models or scenes is not included in our discussion. The computerized reconstruction method may be classified in various ways. It can be classified in various ways according to the use of image and video, the direct use of depth, and the implementation method (rule-based and deep learning-based). In another aspect, the 3D reconstruction method may be divided into static and dynamic paths. Three-dimensional geometric reconstruction of the static environment has been developed in various fields of computer vision and graphics. The most representative study is Simultaneous Localization and Mapping (SLAM) [1,2]. Photogrammetry has also been widely studied and used [3,4,5,6]. Photogrammetry is a measurement technique that uses light rays captured by single or multiple cameras. The technique requires two photographs of the same object captured from different locations. Karami et al. proposed a method for generating an accurate 3D reconstruction of non-collaborative surfaces through a combination of photogrammetry and photometric stereo [3]. Balde et al. proved the feasibility of a 4D monitoring solution (3D modeling and temporal monitoring) for a sandbar and characterized the species’ role in the landscape. The developed solution allowed the study of the interaction between the river dynamics and vegetation using a network of low-resolution and low-power sensors [4]. Ostrowska et al. presented the mapping of fragments of built structures at different scales (finest detail, garden sculpture, architectural interior, building facade) by using a LiDAR sensor from the Apple iPad Pro mobile device. The resulting iPad LiDAR and photogrammetric models were compared with reference models derived from laser scanning and point measurements [5]. Zhan et al. presented a hierarchical image retrieval algorithm based on multiple features and details; the choice of representation of multiple features is critical to the improvement in accuracy of this algorithm using AlexNet-FC7 (fully connected layers) or ResNet101-Pool5 (pooling layers) and local features using SIFT (scale-invariant feature transform) [6]. Similar to photogrammetry, structure from motion (SfM) has been widely researched [7,8,9]. SfM is an image processing technique that has been developed for computer vision applications. The fundamental techniques used in SfM techniques include camera pose estimation, camera calibration, triangulation, and bundle adjustment, which are adapted from photogrammetry. Yin et al. studied a mismatching filtering algorithm based on the local correlation of images in order to get accurate poses. To increase the number of matches, SIFT and ORB feature matching are merged as inputs to sparse reconstruction, and then the incremental SFM algorithm is used to receive sparse 3D points from the picture set used. Finally, they used the combination of optical flow and ORB features to densely reconstruct the image [7]. Shin et al. proposed a robust method in this special environment. Camera parameters were extracted using two types of structure from motion (SfM). Intrinsic camera parameters were extracted via camera calibration, and extrinsic parameters were computed by SfM [8]. Yuan et al. proposed an improved method of 3D scene reconstruction based on SfM. Yuan et al. proposed an improved method of 3D scene reconstruction based on SfM. By taking the video streaming as input, they put forward a feature similarity determination strategy to extract key frames and utilize a dense algorithm to improve the model accuracy. Moreover, the method appends 3D model filtering to remove the redundancy of the resulting models [9].

Along with static components, there are various dynamic objects and scenes in reality. Dynamic objects and scenes have rigid as well as non-rigid surfaces or behaviors. Therefore, dynamic reconstruction should consider more technical factors, such as data fitting, non-rigid registration, strong scene prior, deformable object tracking, etc., than static reconstruction [10,11]. There have also been many studies to analyze and represent smooth (non-rigid) surfaces [12,13,14]. Ge studied the specific context of isometric deformations, which is based on the registration of point clouds at different epochs captured from an isometric deformation surface within overlapping regions. The method shows a success rate for generating true correspondences of 90% and a root mean square error after final registration of 2∼3 mm [12]. Marinov et al. presented a method for scattered data approximation with subdivision surfaces, which actually uses the true representation of the limit surface as a linear combination of smooth basis functions associated with the control vertices [13]. Estellers et al. proposed a model to fit a subdivision surface to input samples that, unlike previous methods, can be applied to noisy and partial scans from depth sensors. The task is formulated as an optimization problem with robust data terms and solved with a sequential quadratic program that outperforms the solvers previously used to fit subdivision surfaces to noisy data [14].

Three-dimensional dynamic reconstruction is a very complex and challenging process. It is still difficult to produce content of a satisfactory grade using an automated method. Therefore, 4D volumetric capture technology that acquires 3D models and scenes for all frames has been studied a lot. The 4D volumetric model is defined as a case in which the 3D volumetric model exists in every frame in time. There are various methods for producing high-quality 3D models in the digital environment. Still, there is a problem: a lot of human resources and time are required to make 3D models fundamentally. To overcome this, various technologies for generating 3D models based on 2D images have emerged, and 4D volumetric capture is attracting attention as the latest model of the technology [15,16,17,18,19]. Guo et al. developed “The Relightables” a volumetric capture system for photorealistic and high-quality relightable full-body performance capture. They presented a new system with a plethora of geometric, lighting, and appearance constraints through the combination of state-of-the-art active illumination, novel high-resolution depth sensors, and a high-resolution camera array [15]. Schreer et al. proposed the production of 360 degree volumetric video for integrated capture and lighting system [16]. They also proposed a complete multi-view 3D processing chain for high-quality sequences of meshes in terms of geometrical detail and texture quality. Chen et al. enhanced a professional end-to-end volumetric video production pipeline to achieve high-fidelity human body reconstruction using only a passive camera [17]. DynamicFusion [19] is a technology for the real-time reconstruction of a 3D model using a depth image captured by a single depth sensor, and the depth information acquired by a single RGB-Depth camera is gradually accumulated.

Four-dimensional volumetric data has the advantage that very high-quality 3D content service is possible by precisely acquiring and storing the shape and motion of a 3D model for every frame. On the other hand, there are disadvantages in that the data capacity is vast, the mesh structure of each frame is not constant, and the texture color according to each frame may be different. We apply a dynamic reconstruction method by gradually accumulating sequences of 3D models generated by volumetric capture. We propose a technique that can create a model of consistent quality over time by interpolating noise information on the surface and correcting the model damaged by occlusion.

This paper is structured as follows. Section 2 introduces the concepts of remeshing and deformation transfer, which are element theories necessary for the development of this paper. Section 3 introduces the algorithm proposed in this paper. Section 4 shows the experimental results, and Section 5 concludes this paper.

## 2. Fundamental Theory

The element technologies of dynamic reconstruction proposed in this paper are remeshing and deformation transfer. In this section, these two principles will be explained first before explaining the proposed approach.

### 2.1. Remeshing

Research on remeshing has been conducted for a very long time and has been conducted in various ways [20]. Studying remeshing or topology aims to reconstruct irregularly structured surfaces into high-quality surfaces. Excellent surface quality can be defined as fidelity, simplicity, and element quality [21]. Fundamentally, a mesh must be able to represent the geometry of an object faithfully. In addition, the number of vertices and the complexity of mesh connections should be reduced for efficient representation and computation. This requires the simplicity of the mesh structure. For the efficient calculation of partial derivatives, integrals, and basis functions on surfaces, well-shaped triangles, that is, triangle meshes with good quality, are required [22]. There are two types of remeshing techniques: a method of generating a mesh structure by modifying the input mesh structure [23] and a method of generating a completely new mesh [24].

Structured remeshing replaces an unstructured input mesh with a structured mesh. Several connecting nodes and faces surround every inner vertex in a structured mesh. Structured meshes offer several advantages over unstructured meshes. The connection graph of a structured mesh is much simpler, allowing efficient navigation and localization. In the sequence of 3D models generated by the photogrammetric method, the remeshing applied in this paper is used to structure the mesh with an irregular structure for each frame, generate a generalized mesh with a similar structure, and obtain a surface with common features between frames.

### 2.2. Deformation Transfer

In 3D computer graphics, animating a target object according to a source animation sequence is a complex problem, and in conventional methods, highly skilled graphic developers have performed this task manually. To solve this problem, deformation transfer (DT) was proposed by Sumner et al. to transfer the motion of the original object to the target object. The DT generates the motion sequence of the target object similar to that of the source object with minimal human intervention. An effective DT should automatically transmit the transformation of the source to the target, and the shape of the transmitted target should be preserved.

Transferring deformations between two different 3D objects is one of the most critical studies in geometry processing. Unlike the case of a rigid surface, which can be easily expressed by rotation and translation, the deformation of a non-rigid surface of a moving object depends on the calculation of the corresponding point or area of the surface between the two objects. In the correspondence of 3D objects, studies that analyze the properties of surfaces using geodesic distance [25], angles of vertices constituting a surface [26], and basis functions [27] based on surface gradient and divergence [27] have been carried out.

When transforming a non-rigid surface, a rigid transform is usually applied to transforms of a small local area. However, when aligned with the object to take the entire surface into account, it transforms in a non-rigid manner. In the case of assigning affine transformations to vertices or deformation graph nodes of a source, regularizations are introduced to make each affine transformation close to a rigid body transformation [28,29,30,31,32,33].

Collet et al. proposed a method to partition the sequence into subsequences to support the deformation of the mesh surface over time [34]. One mesh per subsequence is selected as a key frame, and the selection of key frames identifies similar shapes throughout the sequence. Further, similar frames are registered according to the shortest path through the globally constructed similarity search, and non-rigid transformations are performed non-sequentially [35].

In our paper, the search for the corresponding point of a 3D object is limited to the case where the distance of the elements constituting the surface is preserved even if the object is deformed. For example, in the case of normal joint motion, the movement of the human body is limited to the case where the surface is constantly bent without abnormal deformation, such as torn or stretched. To maintain such isometric characteristics and efficiently search for correspondence points between objects, remeshing is applied to all meshes in the 3D sequence and converted into a typical structure.

## 3. Dynamic Reconstruction of 4D Volumetric Model

This section describes the proposed dynamic reconstruction algorithm.

### 3.1. Overview of the Proposed Algorithm

**Figure 1:**
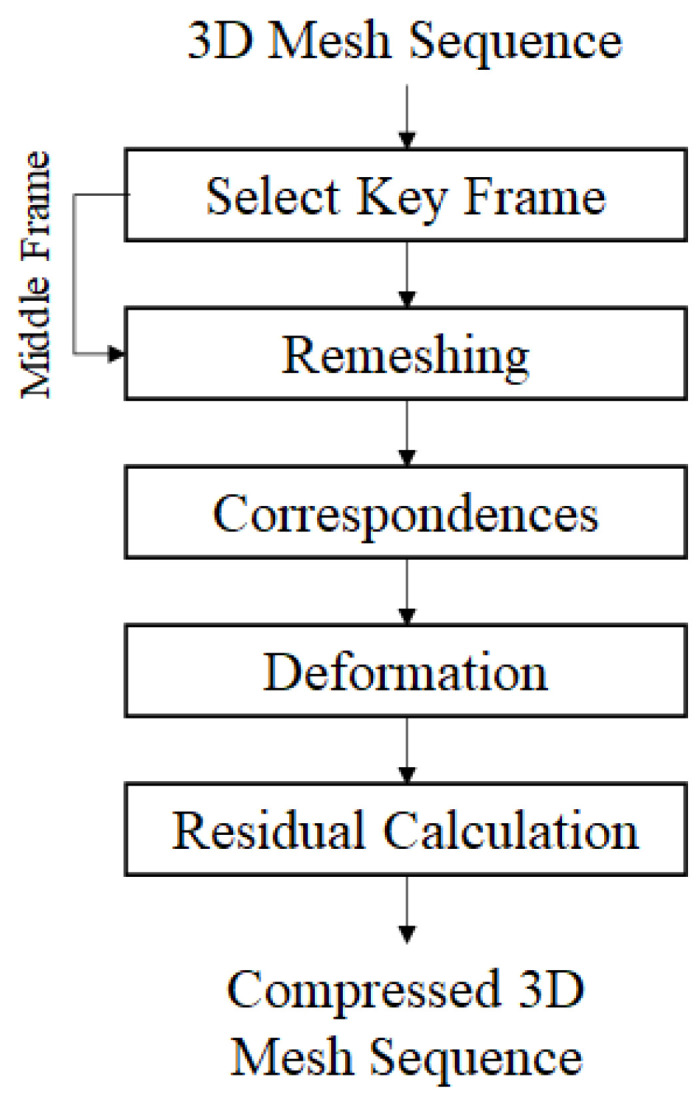
The proposed dynamic reconstruction and compression algorithm.

Figure 1 shows the dynamic reconstruction algorithm of the proposed 4D volumetric model. In a 3D model sequence, the first procedure to obtain information about a moving object and a deformed object is sampling the frames in the sequence at regular time intervals. To compare the 3D model of the target frame and the 3D model of the key frame, a remeshing process is performed for each key frame and each target frame to make the mesh structure of the 3D model similar. This allows the two 3D models to have similar geometries. Next, deformation using the correspondence of the two 3D models is performed. Finally, the two models matched through transformation are updated in the target frame of the current stage. This process is repeatedly performed for all key frames and their target frames. After that, the data compression process is performed by preserving and transmitting only residual information between the transformed key frame and the target frame.

### 3.2. Key Frame Selection

A key frame in a 3D sequence should satisfy the following conditions. The three conditions are;

(1) When more than 15 frames have passed since adding a new key frame.
(2) When the sum of the Euclidean distances between the corresponding points of the key frame and the target frame exceeds 20 cm.
(3) The number of meshes between the key and current frames differs by more than 1000.

If the number of frames differs greatly or the shape changes rapidly, the error rate for the result of deformation may increase significantly. Changes in the number of frames and shapes may depend on the dataset. Therefore, it should be used as a parameter for deformation after experimentally finding a condition in which the error rate rapidly increases. It was experimentally confirmed that the error rate increased by about two times or more when the conditions presented in our dataset were exceeded. These selection conditions are determined experimentally by setting individual parameters.

### 3.3. Remeshing

**Figure 2:**
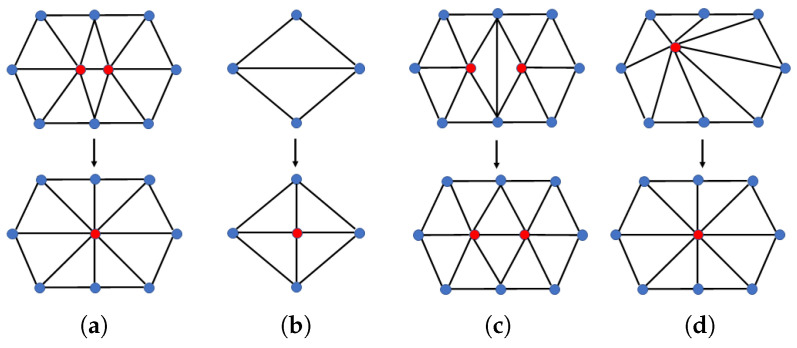
Example of the application of remeshing algorithm. (**a**) Edge collapse, (**b**) edge split, (**c**) edge flip, and (**d**) vertex shift.

In order to structurally remesh the surface, edge collapse, edge split, edge flip, and vertex shift techniques of vertex connection nodes through mesh localization are combined, as shown in Figure 2. In the proposed method, the most important criterion for surface quality is the minimum and maximum angles of the vertices. To calculate the geodesic distance between corresponding points in the key frame deformation step, a mesh structure with only acute triangles is suitable [36,37,38]. Suppose there is an acute angle smaller than the reference or an obtuse angle greater than the reference in the input mesh. In that case, the angle of the triangle is adjusted uniformly using the method in Figure 2. Remeshing is performed on all key frames and target frames.

### 3.4. Correspondence Searching

**Figure 3:**
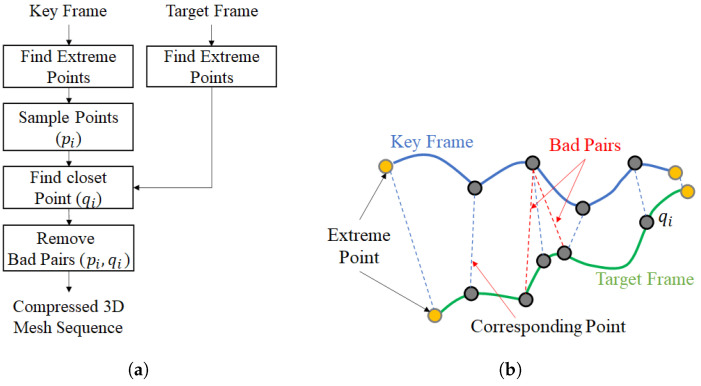
Correspondence point search algorithm. (**a**) The proposed search procedure, (**b**) example of correspondence point searching.

The correspondence searching algorithm is shown in Figure 3. The correspondence of the surfaces of the key frame and target frame $S,\;T\subset R^{3}$ is expressed as f:S→T, and the modified method of the initial correspondence of ICP (Iterative Closest Point) is applied for our method. First, six extreme points of *S* and *T* are defined as the initial correspondence points. The extreme point is a kind of special sample with robust correspondence between two 3D meshes. After the initial correspondence is selected between *S* and *T*, the vertices $p_{i}$ are sampled between the initial correspondence points. Next, the dense correspondence point $q_{i}$ and set ($p_{i}$, $q_{i}$) is calculated by the relationship of correspondence between two surfaces using the sampled vertex $p_{i}$. In this step, the bad pair, which is from an error of connection, may be created. If $q_{i}$ has a connection in the case that $p_{i}$ has multiple connections with $q_{i}$, it is regarded as the bad pair, and it should be removed. Furthermore, if $p_{i}$ does not have any connection between the key and target frames, it leaves as the unconnected point.

### 3.5. Deformation of Key Frame

**Figure 4:**
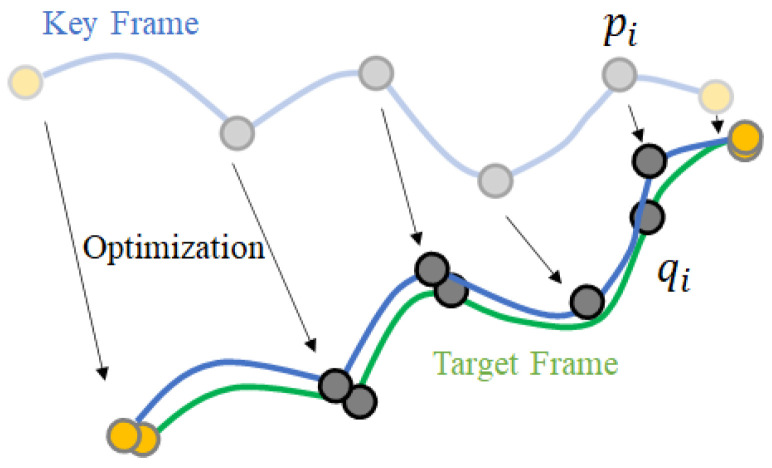
Example of applying key frame deformation.

In the key frame deformation step, the way to align the surface is to minimize the distance between the corresponding points, as shown in Figure 4. By iterative optimization, until this minimization converges, the key frames are progressively deformed into the shape of intermediate frames.

**Figure 5:**
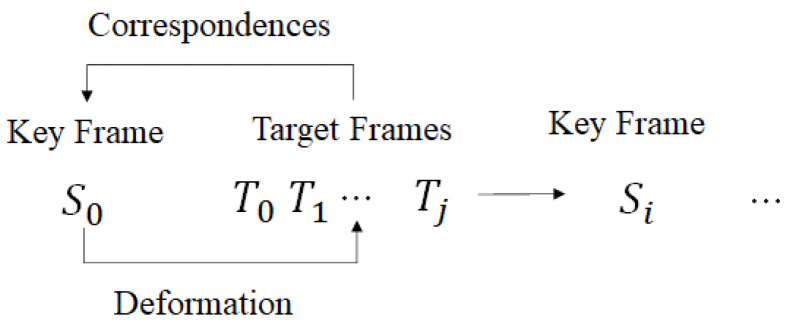
Key frame transformation and update procedure.

Figure 5 shows the update process from the key frame deformation procedure to the target frame. *S* repeats the deformation for all intermediate frames between $T_{j}$ and *S* until $S_{i}$, the next key frame $S_{i}$ appears and updates $S^{{}^{\prime }}$ after the deformation is completed.

## 4. Three-Dimensional Model Compression

The 4D volumetric data have a massive capacity because they have mesh and texture information for the 3D volumetric model for every frame. Therefore, data compression is essential in using volumetric data. To increase the similarity between frames of 4D volumetric data, we proposed a method of deforming key frames to create target frames. A deformed key frame has a shape similar to or identical to the target frame. We use these results to calculate the residual of the target frame and use it as a compression technique. The process for stabilizing 4D volumetric data can be regarded as finding a morphological correlation between temporally defined 3D models.

## 5. Experimental Result

This section presents the experimental results of the proposed dynamic reconstruction method. First, the experimental environment and data used in the experiment will be described. Next, the results of remeshing, matching point search, and deformation are shown. The accuracy is shown through the error in the key frame due to deformation. The performance of the proposed dynamic reconstruction method is shown by comparing the key and target frames. Finally, the result of compressing the 4D volumetric data using dynamic reconstruction is shown.

### 5.1. Environment

**Figure 6:**
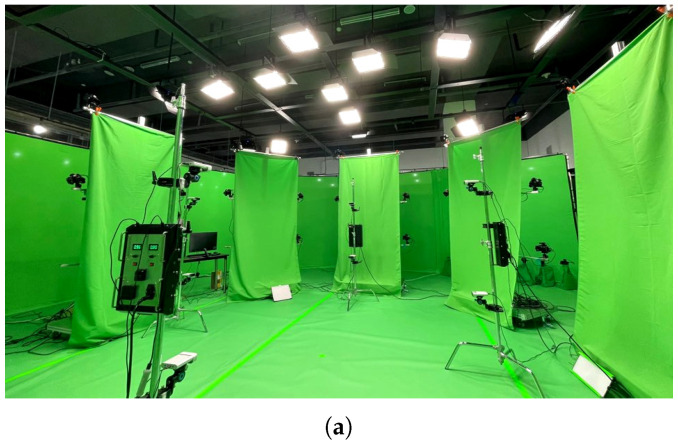

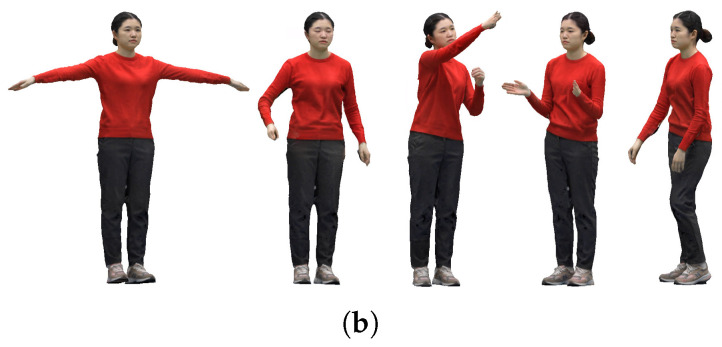
Experimental environment of volumetric capture. (**a**) Four-dimensional volumetric capture studio, (**b**) captured 4D volumetric data.

In the experiment, data of a female model (Sol Lee, the second author) were captured in a volumetric studio using volumetric capture technology. The dataset used in the experiment was photographed using the studio of MnnH Inc. [39], as shown in Figure 6a. The capturing system has 60 high-end cameras with 4K and 8K resolutions, which are made by Sony. The software solution for reconstruction was provided by MnnH Inc. Its shooting range is about 6 m in diameter. As shown in Figure 6b, it was composed of a total of 900 frames and a 30 s 3D model sequence of various motions. The captured volumetric model has about 100,000 meshes per frame, and the resolution of the texture is 4K.

### 5.2. Dynamic Reconstruction

**Figure 7:**
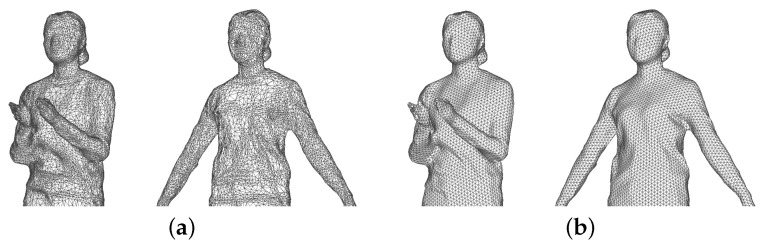
After applying remeshing to the key frame (15 frames) and intermediate frames (30 frames), the mesh structure (**a**) before and (**b**) after application.

Figure 7 shows the results before and after remeshing for key frame and target frame. Compared to the image before remeshing in Figure 7a, the image after remeshing in Figure 7b has a simple surface structure and a triangular structure of even quality close to an equilateral triangle. In addition, the key and target frames are structurally similar and exhibit consistent geometric characteristics.

**Figure 8:**
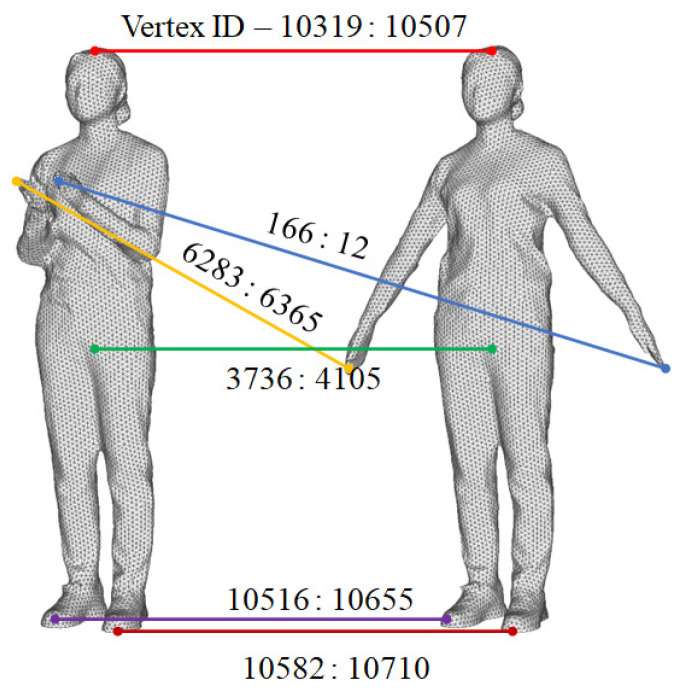
Initial correspondence structure of the key frame (1 frame) and target frame (15 frames).

Figure 8 shows the initial matching point search results of the proposed algorithm using the Sol data set. The poles of each corresponding part are precisely matched. The number shown in Figure 8 is the number of each point in each frame.

**Figure 9:**
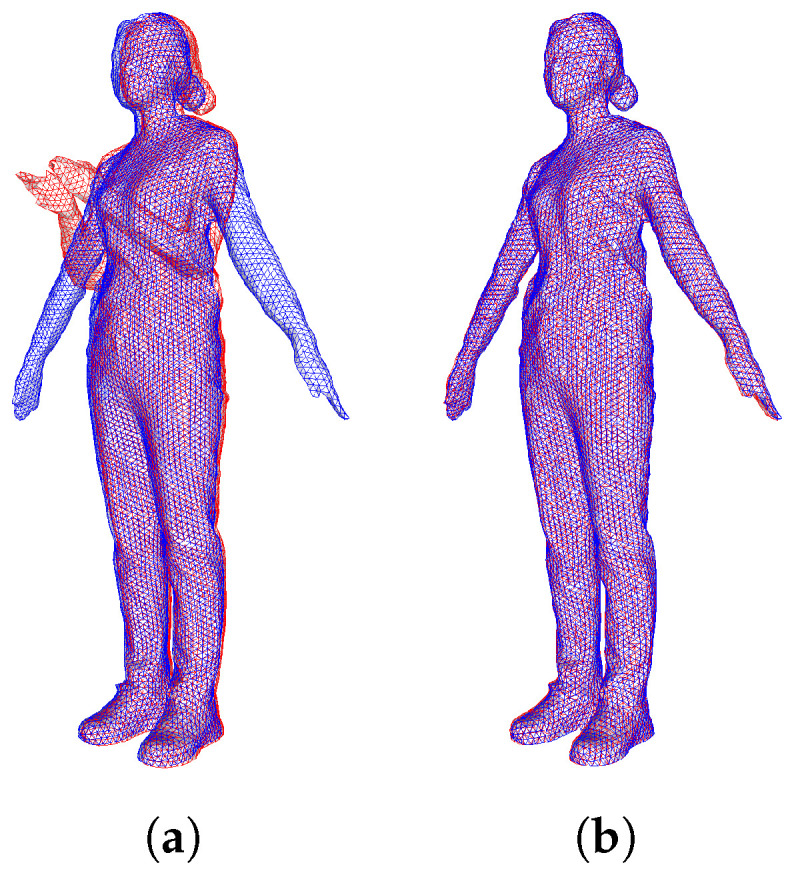
Deformation by calculating the corresponding point between the key frame and the middle frame (**a**) before and (**b**) after deformation.

**Figure 10:**
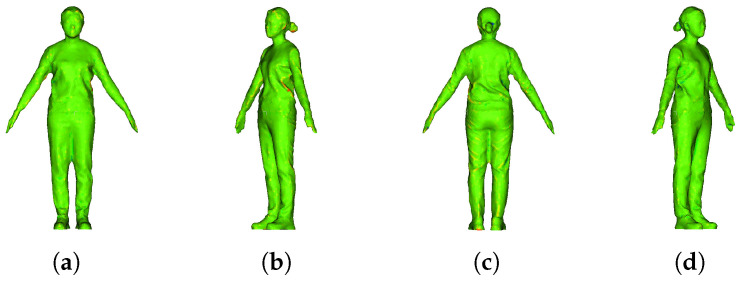
Deformation result by calculating the corresponding point between the key frame and the middle frame. (**a**) Front error map, (**b**) left side error map, (**c**) right side error map, and (**d**) rear error map.

Figure 9 and Figure 10 are the resulting images of the proposed deformation using corresponding points. Figure 9 shows the results before and after applying the deformation of the key frame after remeshing. In Figure 9a,b, the red wireframe represents the key frame mesh, and the blue wireframe represents the target frame mesh. In the resulting image in Figure 9b, the structure of the connection node of the key frame mostly coincides with the middle frame.

### 5.3. Accuracy Analysis

Figure 10 is an image showing the error rate between the key frame and the deformed intermediate frame, and the average and standard deviation of the error were calculated using the error measurement function of CloudCompare [40]. The higher the agreement between the two models, the more green is displayed. The red color is displayed if the surfaces do not match in the positive direction. The mean distance of the two models was 0.23 mm, and the standard deviation was measured to be 0.13 mm.

**Figure 11:**
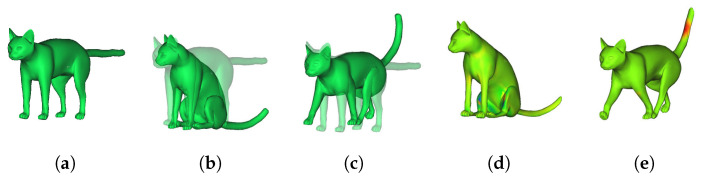
Deformation result by calculating the corresponding point between the key frame and the middle frame. (**a**) a source model, (**b**,**c**) target pose 1 and 2, and (**d**,**e**) error map of pose 1 and 2.

**Figure 12:**
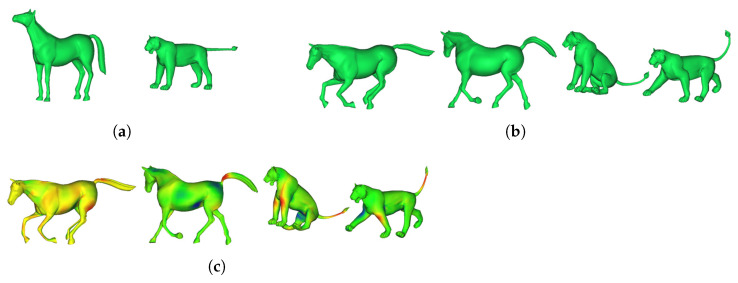
Deformation result by using Horse and Lion of the TOSCA (**a**) source, (**b**) target, and (**c**) error model (error distance = 0.0101, 0.051, 0.0683, and 0.0936 mm, standard deviation of error = 0.412, 0.51, 0.695, and 0.62 mm).

Figure 11 shows the quantitative evaluation results using the Cat among the open data TOSCA dataset [41] to confirm the versatility of the algorithm. First, the 3D model of Figure 11a (corresponding to the key frame) was deformed into the 3D model (corresponding to the target frame) of Figure 11b,c. Next, the error between the two results is displayed as an error map in Figure 11d,e. At this time, the information about the color of each error map is the same as in Figure 10. Figure 12 also shows the deformation results of the Horse and Lion included in the TOSCA dataset. Figure 12a is the source model, and Figure 12b is the target model. We deformed the source model to the target model. The resultant models are shown in Figure 12c. The deformed models in Figure 12c have error distances of 0.0101, 0.051, 0.0683, and 0.0936 mm and standard deviation of 0.412, 0.51, 0.695, and 0.62 mm.

**Figure 13:**
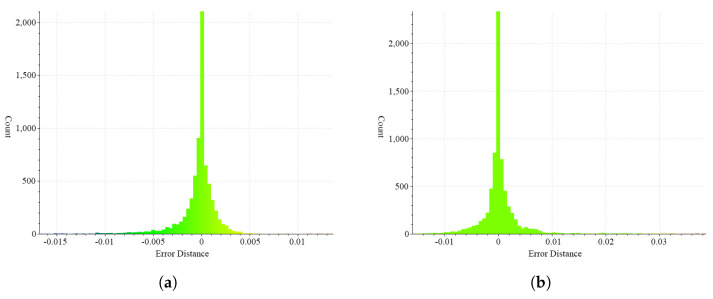
Deformation application result by calculating the corresponding point between the key frame and the middle frame of dataset 2 (**a**) surface structure, (**b**) error map.

Figure 13 expresses the difference between the deformed surface and the original surface as a histogram. The error in Figure 11d is shown in Figure 13a, and the error in Figure 11e is shown in Figure 11b. The mean error of pose 1 is 0.0352 mm, and the standard deviation is 0.2022 mm. The mean error of Pose 2 is 0.0995 mm, and the standard deviation is 0.4060 mm.

The results for the TOSCA Cat were compared with those of previous studies. The comparison results are shown in Table 1. In Table 1, the average error of the nine movements of the TOSCA Cat is 0.06mm. The error was improved by about 98.88% compared to Xuming [12], 22.23% compared to Marinov’s study [13], and 20.39% compared to Estellers’ study [14]. Table 1 also shows the comparisons of the processing time. As can be expected, in general, the processing time increases as the complexity of the algorithm increases. Our algorithm has the highest complexity and takes about 1.6 times more time than the result of Xuming.

**Figure 14:**
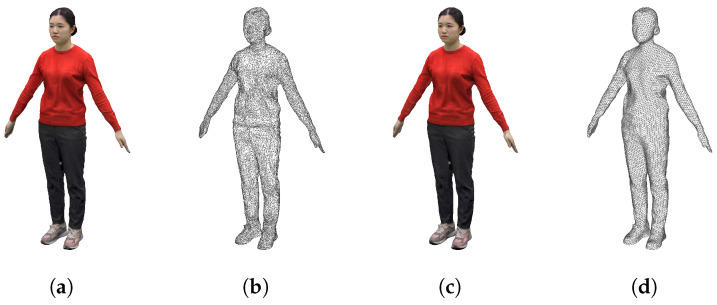
Comparison of the original and deformed mesh of key and target frames after remeshing. (**a**) Original texture of target frame, (**b**) original mesh of target frame, (**c**) transferred result of texture, and (**d**) deformed mesh of key frame.

Figure 14 is the final image of transmitting the texture of the target frame after key frame deformation. Each connection node has a simplified and regular structure compared to the original. When checking the quality of the shredded texture, the texture remained almost identical with no distortion.

### 5.4. Compression Result

**Figure 15:**
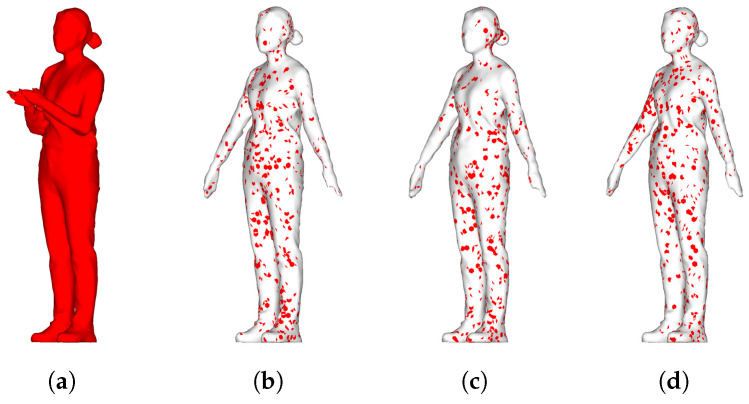
Residual data comparison image between the original key frame and the transformed intermediate frame. (**a**) key frame, (**b**) A, (**c**) B, and (**d**) C target frames.

Figure 15 shows the result of deforming the original key frame to the target frames A, B, and C for compression of 4D volumetric data and obtaining the residual. In Figure 15, the red spots correspond to the residual mesh. The results of Table 2 showed a compression efficiency of 50% in the remeshing process when compared with the capacity of the original key frame. Next, the capacity of each target frame A, B, and C was reduced to 7.46%, 8.36%, and 7.46% in calculating the deformation and residuals. Considering the entire sequence, the data could be compressed to a size of 18.48% of the original sequence capacity.

## 6. Conclusions

This paper proposes a dynamic reconstruction algorithm for the non-rigid deformation of a mesh surface using correspondence for processing 4D volumetric data. The proposed algorithm was verified using a 4D volumetric model consisting of 900 frames. This volumetric model has about 100,000 meshes per frame, and the texture resolution is 4K. The mean distance of the dynamic reconstruction result of the volumetric model we captured was 0.23 mm, and the standard deviation was 0.13 mm, showing high accuracy. Furthermore, compared with previous studies using the TOSCA Cat, the proposed method showed improved error rates of up to 98.88% and at least 20.39% compared to previous studies. Finally, when the proposed algorithm is used to compress a 4D volumetric sequence, data can be compressed to 18.48% of the original sequence capacity without using a video codec. Based on these results, we intend to study the deformation of non-rigid objects with very high complexity. Research on very delicate, non-rigid deformation, such as fine changes in clothes and fine wrinkles on the face, will play a very important role in the field of computer vision in the future.

## Figures and Tables

**Table 1:** Numerical comparison for the accuracy of the TOSCA Cat by using CloudCompare.

Method	Error Distance	Error Ratio	Processing Time	Time Ratio
Xuming [12]	1.17 mm	0.00%	312 ms	0.00%
Marinov et al. [13]	0.32 mm	72.65%	431 ms	138.14%
Estellers et al. [14]	0.24 mm	79.49%	445 ms	142.63%
Ours	0.06 mm	94.88%	520 ms	166.67%

**Table 2:** Compression result of the 4D volumetric data.

		Key Frame	Target Frame			Total
			Frame A	Frame B	Frame C	
Raw	Vertices	19,522	19,249	19,013	19,999	77,783
	Face	39,040	38,494	38,022	39,994	155,550
	Byte (MB)	3.4	3.35	3.3	3.49	13.54
Remesh	Vertices	10,583	10,421	10,324	10,712	42,040
	Face	21,162	20,838	20,644	21,420	84,064
	Byte (MB)	1.7	1.68	1.67	1.73	6.78
Residual	Vertices	10,583	4749	4828	5011	25,171
	Face	21,162	6032	6420	6618	40,232
	Byte (MB)	1.7	0.25	0.28	0.27	2.5
Ratio (%)	Vertices	54.21%	24.67%	25.39%	25.06%	32.36%
	Face	54.21%	15.67%	16.88%	16.55%	25.86%
	Byte (MB)	50.00%	7.46%	8.39%	7.68%	18.43%

## Data Availability

Not applicable.
